# Therapeutic potential of new B cell-targeted agents in the treatment of elderly and unfit patients with chronic lymphocytic leukemia

**DOI:** 10.1186/s13045-015-0165-x

**Published:** 2015-07-14

**Authors:** Kanti R. Rai

**Affiliations:** Hofstra North Shore-LIJ School of Medicine and the North Shore-LIJ Cancer Institute, Lake Success, NY USA; CLL Research & Treatment Program, NSLIJ Health System, Suite 212, 410 Lakeville Road, New Hyde Park, NY 11042 USA

**Keywords:** BTK, Chronic lymphocytic leukemia, Comorbidities, Elderly, PI3K

## Abstract

Chronic lymphocytic leukemia (CLL), the most common adult leukemia in the Western world, is primarily a disease of the elderly, with most patients ≥65 years of age and having at least one major comorbidity. Aggressive chemoimmunotherapy regimens recommended to achieve remission and improve survival in young, fit patients are often poorly tolerated in elderly and/or less physiologically fit (“unfit”) patients, necessitating alternative treatment options. Although patient age, fitness, and comorbidities are key considerations in the selection of a treatment regimen, historically, clinical trials have been limited to young, fit patients by virtue of the ethical concerns associated with potential end organ toxic effects that could worsen comorbidities. However, the availability of new therapies promises a shift to a research paradigm that encompasses the identification of optimal treatments for elderly and unfit patients. Anti-CD20 monoclonal antibody therapy, which overall has improved response rates and survival in patients with CLL, has only recently been evaluated elderly and unfit patients. B cell-targeted agents such as the Bruton’s tyrosine kinase inhibitor ibrutinib and the phosphatidylinositol 3-kinase inhibitor idelalisib are the first of a new generation of oral agents for CLL. Available clinical data suggest that these therapies have the potential to address the unmet need in elderly and unfit patients with CLL and result in clinical remission, and not merely symptom palliation and improved quality of life, which, by themselves, are also a reasonable goal.

## Introduction

**Fig. 1:**
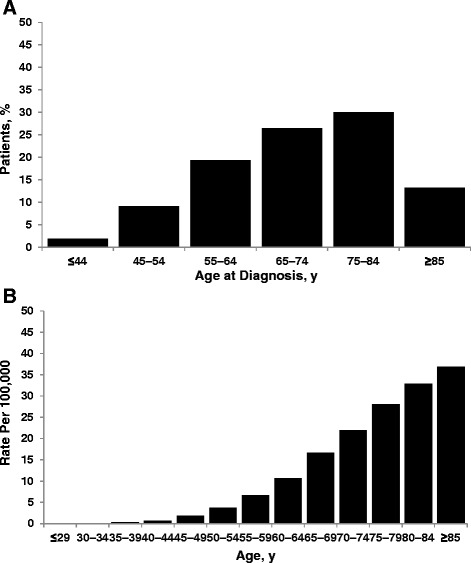
Age-related CLL statistics in the USA. **a** Percentage of US patients by age at CLL diagnosis, 2009 [6]. **b** Age-specific incidence rates of CLL, 2007–2011 [7]. *CLL* chronic lymphocytic leukemia

**Fig. 2:**
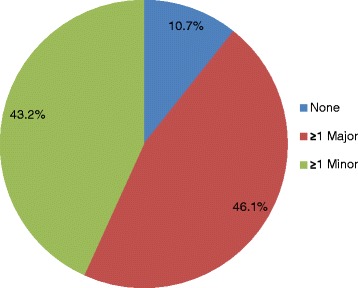
Comorbidities in patients with CLL [8]. Major comorbidities include cardiac disease, diabetes mellitus, respiratory disease, and other malignancy. *CLL* chronic lymphocytic leukemia

Chronic lymphocytic leukemia (CLL) is a lymphoproliferative disorder whose clinical features include the abnormal proliferation of mature B cells in peripheral blood, bone marrow, and lymph nodes [1]. It is the most common adult leukemia in the Western world [2]; in the USA, approximately 15,720 new CLL cases and 4600 deaths are expected to have occurred in 2014 [3]. CLL is primarily a disease of the elderly, with a median age at diagnosis of approximately 72 years [4, 5] and nearly 70 % of diagnoses in patients ≥65 years of age (Fig. 1a) [6]. The incidence of CLL increases progressively with each decade in patients >60 years of age (Fig. 1b) [7], and most patients have at least one major comorbidity (Fig. 2) [8].

Chronic lymphocytic leukemia is currently considered incurable [1], but in many patients, the disease is indolent. Therefore, even though diagnosis is typically made early in the disease course [1], therapy is reserved for those with advanced, symptomatic, or aggressive disease [9]. Accordingly, patients typically receive their first treatment at an older age [6], when they may be frail and have comorbidities that complicate treatment.

Within the current CLL treatment paradigm, there are important unmet needs in elderly and less physiologically fit (unfit) patients. This article reviews the evolution and current status of therapy for CLL, with particular regard to elderly and unfit patients, and discusses the potential of B cell-targeted agents.

### Current CLL treatment paradigm

The clinical course of CLL is heterogeneous [1], and after a diagnosis is made, staging and prognostic assessment are important to determine the anticipated disease course and appropriate therapy, if any [1, 10]. Prognostic factors include basic laboratory parameters (e.g., absolute lymphocyte count, lymphocyte doubling time, serum lactate dehydrogenase), immunoglobulin heavy chain status, and cytogenetic profile (e.g., del 13q, del 11q, del 17p, and trisomy 12 status) [1, 11]. Patient characteristics, including age, fitness, functional status, and comorbidities, are equally important [1, 10, 12]. In relapsed patients, response to first-line treatment should also be taken into consideration [12].

These principles are reflected in the National Comprehensive Cancer Network (NCCN) Clinical Practice Guidelines (Table 1) [10]. In younger and/or fit patients with CLL, the goal is to achieve complete remission and prolong survival [6], and the NCCN guidelines recommend chemoimmunotherapy as first-line treatment. The combination of with fludarabine, cyclophosphamide, and rituximab (FCR) was the first therapy demonstrated to prolong overall survival in patients with CLL [13] and is the current standard of care [10, 14]. In relapsed/refractory patients, treatment is guided by the length of response to first-line treatment. In patients who had a long response, it is recommended that first-line treatment be repeated until a short response is obtained, whereas in patients who had a short response, second-line treatment with ibrutinib, idelalisib ± rituximab chemoimmunotherapy, ofatumumab, obinutuzumab, lenalidomide ± rituximab, alemtuzumab ± rituximab, or high-dose methylprednisolone + rituximab is recommended [10].

**Table 1:** NCCN-suggested treatment regimens^a^ for CLL [10]

Setting	Younger/fit patients^b^	Elderly/physiologically unfit patients^c^
First-line therapy	Chemoimmunotherapy	Obinutuzumab + chlorambucil
	FCR	Ofatumumab + chlorambucil
	FR	Rituximab + chlorambucil
	PCR	Bendamustine ± rituximab
	Bendamustine ± rituximab	Obinutuzumab
		Fludarabine ± rituximab
		Chlorambucil
		Rituximab
		Cladribine
Relapsed/refractory therapy (short response)^d^	Ibrutinib	Ibrutinib
	Idelalisib ± rituximab	Idelalisib ± rituximab
	Chemoimmunotherapy	Chemoimmunotherapy
	FCR	Reduced-dose FCR
	PCR	Reduced-dose PCR
	Bendamustine ± rituximab	Bendamustine ± rituximab
	Fludarabine ± alemtuzumab	HDMP + rituximab
	RCHOP	Rituximab + chlorambucil
	OFAR	Ofatumumab
	Ofatumumab	Obinutuzumab
	Lenalidomide ± rituximab	Lenalidomide ± rituximab
	Alemtuzumab^e^ ± rituximab	Alemtuzumab^e^ ± rituximab
	HDMP + rituximab	Dose-dense rituximab

*CLL* chronic lymphocytic leukemia, *FCR* fludarabine, cyclophosphamide, and rituximab, *FR* fludarabine and rituximab, *HDMP* high-dose methylprednisolone, *NCCN* National Comprehensive Cancer Network, *OFAR* oxaliplatin, fludarabine, cytarabine, and rituximab, *PCR* pentostatin, cyclophosphamide, and rituximab, *RCHOP* rituximab, cyclophosphamide, doxorubicin, vincristine, and prednisone

^a^CLL without del 11q or del 17 p; regimens are listed in order of preference

^b^Age <70 years, or older patients without significant comorbidities

^c^Age ≥70 years, or younger patients with comorbidities

^d^In patients with long response, suggested to re-treat as in first-line therapy until short response

^e^Alemtuzumab is no longer commercially available for CLL

Because aggressive therapy is often poorly tolerated by older patients and patients who are less physiologically fit [15], for patients ≥70 years of age or younger patients with significant comorbidities, the NCCN guidelines recommend alternative chemoimmunotherapies such as obinutuzumab + chlorambucil and rituximab + chlorambucil as first-line treatment [10]. Similarly, in relapsed/refractory patients, alternatives such as reduced-dose FCR and reduced-dose pentostatin with cyclophosphamide and rituximab are recommended. The goals of these less aggressive treatment regimens are to achieve symptom palliation and maximize quality of life [16]. Obtaining high rates of complete response (CR) in these patients may necessitate new treatment approaches.

### Anti-CD20 monoclonal antibody therapy for CLL

Large randomized trials demonstrated significant improvement in overall response rate and progression-free survival (PFS) with addition of rituximab to fludarabine and cyclophosphamide (FCR regimen) in patients with previously untreated CLL [13] and relapsed CLL [17]. However, patients in these studies were relatively young (median age, 61 [13] and 63 years [17]) and fit, such that benefit in elderly/unfit patients could not be established.

There are few trials in elderly patients with CLL [6], but some data support the applicability of rituximab-based regimens in this population. A recent study of rituximab added to an oral low-dose FC regimen in 30 elderly patients (median age, 75 years) reported a CR of 80 % in frontline patients and 30 % in previously treated patients, with only mild hematologic toxicity [18]. A larger study is needed to confirm these promising findings. In a retrospective study in outpatients ≥70 years of age receiving bendamustine monotherapy versus rituximab + bendamustine, the overall response rate was 50 % versus 67 %, respectively, in treatment-naive patients and 45 % versus 64 % in relapsed/refractory patients, with no unexpected toxicities [19]. Results of a subgroup analysis of a phase 2 clinical trial suggest that rituximab in combination with pentostatin and cyclophosphamide is effective and tolerable in older (≥70 years) as well as younger patients [20].

Compared with data from historical trials of rituximab-based chemoimmunotherapy (rituximab with pentostatin and cyclophosphamide), recent data for ofatumumab-based chemoimmunotherapy (ofatumumab, pentostatin, and cyclophosphamide) are favorable with regard to efficacy and hematologic toxicity in patients with previously untreated CLL (37.5 % ≥70 years) [21]. However, randomized trials comparing these regimens are currently lacking.

Obinutuzumab (formerly GA101) is a glycoengineered type 2 antibody that, like rituximab, kills cells via binding to the CD20 antigen. In patients with previously untreated CLL and coexisting conditions, most of whom were >70 years of age, obinutuzumab + chlorambucil chemoimmunotherapy significantly improved outcomes (PFS; overall, complete, and molecular response; and overall survival) compared with rituximab + chlorambucil [22]. In November 2013, the US Food and Drug Administration approved obinutuzumab for use in combination with chlorambucil in patients with previously untreated CLL.

### Unmet needs in elderly and unfit patients with CLL

Comorbidity, more than age, limits the use of aggressive chemoimmunotherapy in CLL [6]. However, there is no standard method to define patient fitness [6]. Eastern Cooperative Oncology Group (ECOG) [23] and Karnofsky performance [24] status, although they lack components to adjust for specific comorbidities, are widely used in the USA and as entry criteria in CLL clinical trials. NCCN guidelines recommend that assessment of fitness consider age and performance status as well as comorbidities, which can be evaluated using tools and scoring systems such as the Charlson Comorbidity Index (CCI), Cumulative Illness Rating Scale (CIRS), or National Cancer Institute (NCI) Comorbidity Index [10]. The CCI derives a total score based on the presence (0 = absent; 1 = present) and severity (1 = not ill; 5 = moribund) of 30 comorbid diseases [25]. It has been used to predict mortality risk in a variety of medical conditions [26], and Sorror and colleagues [27] modified the CCI to develop a hematopoietic cell transplantation-specific comorbidity index. The National Institute on Aging and NCI comorbidity index was developed using the NCI Surveillance, Epidemiology, and End Results (SEER) program registries to assess comorbidity prevalence in patients with cancer ≥65 years of age [28, 29]. It includes 24 major comorbidity categories rated on a 4-point severity scale (1 = current medical management or diagnostic problem; 4 = condition noted; unknown if history or current) [29]. The CIRS is an instrument for rating physical impairment across 6 bodily systems and 13 organ areas using a 5-point severity scale (0 = none; 4 = extremely severe) and can be used to assess current or cumulative illness [30]. Last, the Index of Coexistent Disease (ICED) measures comorbidity severity (5-point scale) across 14 disease categories and related physical impairment (4-point scale) within 10 functional areas [31]. The Sorror versions of the CCI and the ICED are considered most applicable to the CLL population, although validation in CLL is still lacking [6]. Irrespective of the method for defining patient fitness, knowledge of the presence of comorbidities should be paired with clinical judgment to help guide treatment decisions in elderly patients with CLL.

There is a clear need for greater representation of elderly, unfit patients in randomized clinical trials of CLL chemotherapy [32] to assess the therapeutic endpoint, CR, in this key population. As noted in the NCCN guidelines, the German CLL Study Group trial of first-line treatment with fludarabine versus chlorambucil in patients >65 years of age [33] is the only completed phase 3 trial that has specifically enrolled elderly patients with CLL.

### Potential role of B cell-targeted agents in CLL

**Fig. 3:**
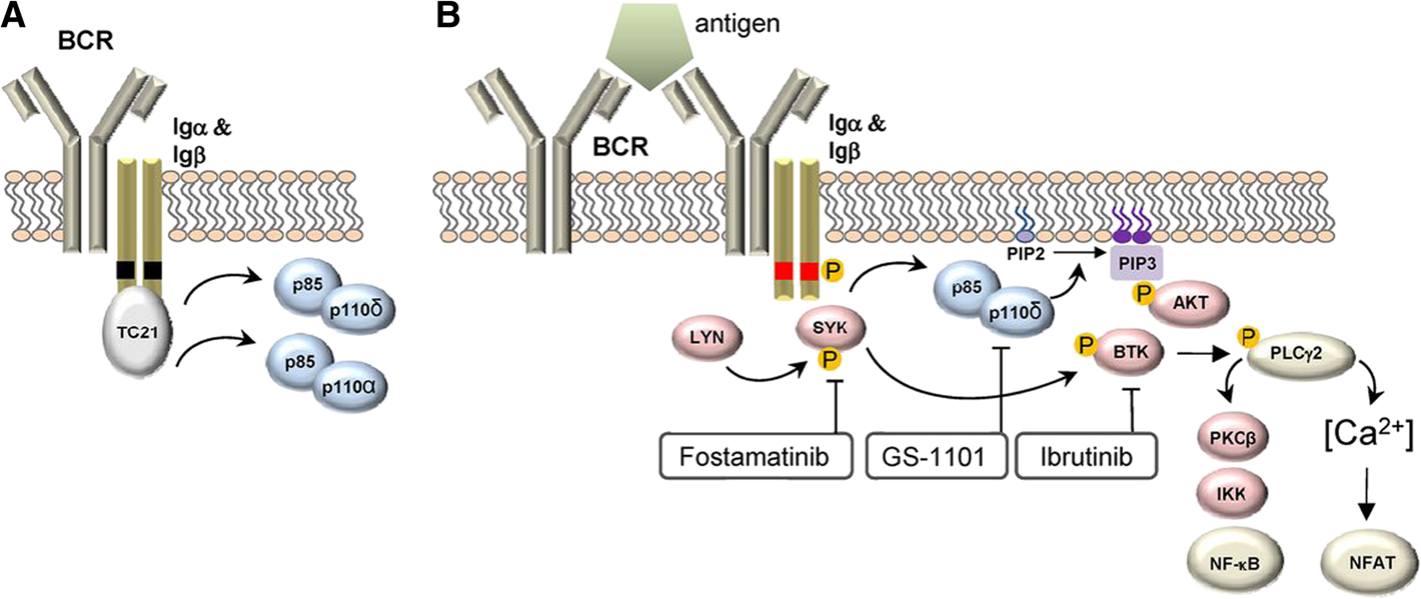
BCR signaling cascades in the **a** absence and **b** presence of antigen. Protein kinases are shown in *red* and the lipid kinase PI3Kδ in *blue* [38]. *BCR* B-cell receptor, *BTK* Bruton’s tyrosine kinase, *Ig* immunoglobulin, *IKK* I kappa B kinase, *NF* nuclear factor, *NFAT* nuclear factor of activated T cells *P* phosphorylation, *PI3Kδ* phosphatidylinositol 3-kinase, *PKC* protein kinase C, *PLC* phospholipase C, *SYK* spleen tyrosine kinase. From [38]

Therapy for CLL is evolving toward targeted approaches firmly grounded in an understanding of the disease pathophysiology [34, 35]. The B-cell receptor (BCR) regulates fundamental B-cell processes, including resting homeostasis, differentiation, proliferation, and survival [36]. Ablation of the BCR leads to rapid B-cell apoptosis, suggesting that it confers a survival signal to B cells [37]. These functions of the BCR are mediated by tonic and antigen-induced signals, which are transmitted by various kinases, including Lyn kinase, spleen tyrosine kinase (SYK), phosphatidylinositol 3-kinase (PI3K), and Bruton’s tyrosine kinase (BTK), via intracellular signaling cascades (Fig. 3) [38].

Bruton’s tyrosine kinase, which is overexpressed in CLL [39], initiates a signaling cascade leading to the activation of the transcription factors nuclear factor kappa B and nuclear factor of activated T cells [38]. These transcription factors play an essential role in B-cell proliferation [40] and survival [41].

PI3K, a phosphatidylinositol lipid kinase, phosphorylates lipid second messengers, leading to the recruitment of a variety of effectors involved in the growth, proliferation, differentiation, and survival of B cells [42, 43]. Notably, survival of resting, mature B cells is dependent on a BCR signal mediated by PI3K [44]. PI3K is more active in B cells of patients with CLL compared with those of healthy persons [45].

Spleen tyrosine kinase is activated upstream of BTK and PI3K and has cooperative and overlapping functions with respect to these kinases, including activation of phospholipase C gamma 2, an important effector [46]. Downstream effects include the regulation of B-cell activation, growth, differentiation, and maturation [46].

Modulation of these kinases offers a potential strategy to address the various perturbations of BCR signaling in CLL [36, 47]. Ibrutinib (PCI-32765) is an oral, first-in-class BTK inhibitor indicated for the treatment of CLL in patients who have received ≥1 prior therapy. In preclinical in vitro and in vivo studies, ibrutinib inhibited BCR-controlled integrin activation, BCR-activated chemokine release, and the adhesion, migration, and survival of CLL cells [48, 49]. Idelalisib (CAL-101) is a first-in-class PI3K inhibitor indicated for the treatment of relapsed CLL, in combination with rituximab, in patients for whom rituximab alone would not be considered appropriate therapy because of other comorbidities. It is selective for the PI3Kδ isoform [45], which is generally expressed only in leukocytes [50]. In vitro, idelalisib inhibited the tonic PI3K survival signal and induced apoptosis in multiple B-cell malignancies [45]. It exhibited dose-dependent cytotoxicity in CLL cells, while sparing T cells and natural killer cells [45]. These data provide a potential mechanism of action for clinical activity in CLL without toxicities associated with off-target effects in nonhematopoietic cells [45]. Fostamatinib is an oral SYK inhibitor in development for B-cell non-Hodgkin lymphoma (NHL) and CLL [51]. In primary tumor cells of patients with relapsed CLL, fostamatinib reduced BTK phosphorylation, activation of downstream effectors, and CLL cell activation and proliferation [52]. Other kinase inhibitors in clinical development for CLL include the PI3K inhibitors TGR-1202 and IPI-145 [53, 54], the BTK inhibitors CC-292 and ONO-4059 [55, 56], the SYK inhibitor GS-9973 [57], and the multikinase inhibitor dasatinib [58].

An additional investigational molecular target for CLL therapy is BCL-2, a protein that protects cells from apoptosis [59]. Inhibition of BCL-2 would allow for programmed cell death. ABT-199, a first-in-class BCL-2-selective inhibitor [59], induced apoptosis in CLL cells [59] and had cell-killing activity in NHL cell lines [60] while sparing platelets [59, 60]; it also promoted tumor regression in mouse xenograft models [60]. Preliminary data in three patients with refractory CLL show rapid tumor lysis [60].

### Raising the bar: potential to elevate treatment goals beyond palliation specifically in the elderly and unfit

Numerous clinical trials in elderly and unfit patients with CLL are currently ongoing (Table 2). Treatments include monotherapy (e.g., kinase inhibitor, anti-CD20 monoclonal antibody) and combination therapy (e.g., kinase inhibitor ± chemotherapy, kinase inhibitor ± anti-CD20 monoclonal antibody, anti-CD20 monoclonal antibody ± chemotherapy). The majority of these trials are in previously untreated patients. Results from two trials of B cell-targeted agents (one trial of ibrutinib and one of idelalisib) in elderly or unfit patients have been reported.

**Table 2:** Ongoing key clinical trials in elderly or unfit patients with CLL

Study	Design	Treatment	Patients	Primary outcome
NCT01722487	Phase 3, randomized, multicenter, open-label (RESONATE-2)	Ibrutinib vs chlorambucil	Frontline CLL/SLL, ≥65 years	PFS
NCT01886872	Phase 3, randomized, multicenter, open-label	Ibrutinib vs ibrutinib + rituximab vs bendamustine + rituximab	Frontline CLL, ≥65 years	PFS
NCT01203930	Phase 2, single-arm, multicenter, open-label	Idelalisib or idelalisib + rituximab	Frontline CLL/SLL, ≥65 years	OR
NCT00645606	Phase 3, randomized, multicenter, open-label	Rituximab vs observation as maintenance after FCR	Frontline CLL, >65 years	PFS
NCT01263704	Phase 2, single-arm, multicenter, open-label	Rituximab + low dose fludarabine + cyclophosphamide	Frontline CLL, ≥65 years	OR
NCT01832922	Phase 1, nonrandomized, multicenter, open-label, dose-ranging	Bendamustine + rituximab	CLL/SLL, multiple comorbidities ± renal insufficiency	AEs, MTD
NCT02015208	Phase 1/2, single-center, single-arm, open-label	Ruxolitinib	Frontline CLL, ≥65 years or ≥18 years with 17p deletion	OR
NCT01444716	Phase 2, single-center, single-arm, open-label	Ofatumumab	Frontline CLL, ≥65 years, unfit	OR
NCT01809847	Phase 2, multicenter, single-arm, open-label	Ofatumumab + dexamethasone (induction) and ofatumumab (maintenance)	Poor-risk CLL, >55 years	OR, rate of MRD-negative status

*AE* adverse event, *CLL* chronic lymphocytic leukemia, *CR* complete response, *FCR* fludarabine, cyclophosphamide, rituximab, *MRD* minimum residual disease, *MTD* maximum tolerated dose, *OR* overall response, *OS* overall survival, *PFS* progression-free survival, *PS* performance status, *SLL* small lymphocytic lymphoma

### First-line ibrutinib therapy

A phase 1b/2, open-label, US, multicenter trial evaluated the clinical safety and efficacy of ibrutinib as first-line treatment in patients ≥65 years of age with CLL or small lymphocytic lymphoma (SLL) [61]. Patients received 28-day cycles of once-daily ibrutinib 420 or 840 mg, but the 840-mg cohort was closed because of demonstration of comparable activity of the doses in another study. The primary endpoint was safety, assessed by adverse events (AEs) and the study design was such that validation of safety endpoints would result in study termination. The proportion of patients that achieved an overall response, PFS, and the long-term tolerability and pharmacodynamics of ibrutinib were secondary endpoints.

**Fig. 4:**
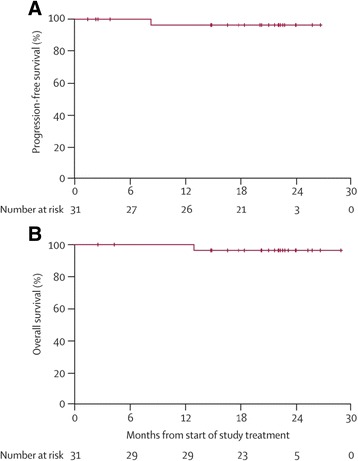
Phase 1b/2 trial of ibrutinib monotherapy in elderly patients with CLL/SLL [61]. **a** Progression-free survival. **b** Overall survival. + = censored. *CLL* chronic lymphocytic leukemia, *SLL* small lymphocytic lymphoma. Reproduced with permission from [61]

A total of 31 patients (CLL, *n* = 29) were enrolled, with a median age of 71 years; 74 % of patients were >70 years of age. Median time from initial diagnosis to study entry was 57.3 months; based on ECOG performance status (0, 74 %; 1, 26 %), patients were relatively fit, despite having several comorbidities. Median treatment duration was 21.0 months, during which relative dose intensity was 98.9 %. The most common overall AEs were diarrhea (68 %), nausea (48 %), and fatigue (32 %), and the most common grade 3 AEs were diarrhea (13 %) and hypertension (6 %). There was one grade 4 AE (thrombocytopenia) and no grade 5 AEs. Two patients discontinued because of AEs (grade 3 fatigue, grade 2 viral infection). The overall response rate was 71 %, with 13 % of patients achieving a CR. Exploratory subgroup analyses showed no differences in overall response, including in patients ≥70 and <70 years of age and in patients with and without high-risk cytogenetics. At 24 months, PFS and overall survival were 96.3 and 96.6 %, respectively. Median PFS was not reached, as only one patient progressed during the follow-up period (Fig. 4). Twenty-six patients (84 %) continued in the optional long-term extension study, the results of which have not yet been reported.

Two phase 3, randomized, open-label trials of ibrutinib in patients ≥65 years of age are currently ongoing (Table 2). These include a trial of rituximab + bendamustine, rituximab + ibrutinib, and ibrutinib monotherapy in patients with previously untreated CLL (NCT01886872) and a multicenter trial of ibrutinib versus chlorambucil in patients with previously untreated CLL or SLL (NCT01722487).

### Idelalisib + rituximab in previously treated CLL

A pivotal phase 3, randomized, double-blind, placebo-controlled trial was undertaken to evaluate the safety and efficacy of idelalisib + rituximab versus placebo + rituximab in previously treated patients with progressive CLL who were unsuitable for cytotoxic therapy (decreased renal function, previous therapy-induced myelosuppression, or major coexisting illness CIRS score >6 for illnesses unrelated to CLL) [62]. Patients were stratified based on deletion/mutation status and randomly assigned to treatment with idelalisib (150 mg twice daily) + rituximab or placebo + rituximab.

**Fig. 5:**
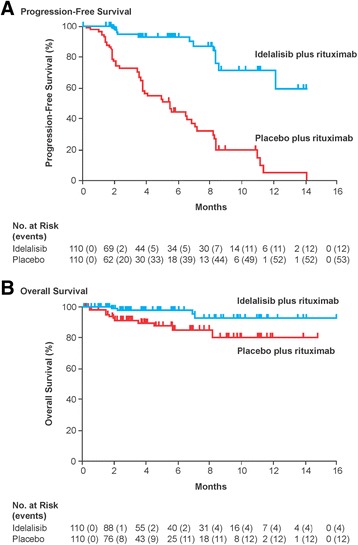
Phase 3 trial of idelalisib + rituximab versus rituximab monotherapy in patients with relapsed CLL and clinically significant coexisting medical conditions [62]. **a** Progression-free survival. **b** Overall survival. *CLL* chronic lymphocytic leukemia. Reproduced with permission from [62]

Of the 220 patients enrolled, 78 % were ≥65 years of age, 85 % had a CIRS score >6 (median score, 8), and 40 % had at least moderate renal dysfunction; high-risk cytogenetics were common. Patients had received a median of three prior regimens. Median on-study treatment duration was 3.8 months for the idelalisib group and 2.9 months for placebo (not significant). At 24 weeks, PFS, the primary endpoint, was significantly higher in the idelalisib group compared with the placebo group (93 vs 46 %; hazard ratio (95 % CI), 0.15 (0.08–0.28); unadjusted *P* < 0.001). Median PFS was not reached in the idelalisib group, whereas it was 5.5 months in the placebo group (Fig. 5a). The treatment effect was similar in all prespecified subgroups, including patients <65 versus ≥65 years of age and those with high-risk cytogenetics and without high-risk cytogenetics. The idelalisib group also demonstrated significant improvement versus placebo in the overall response rate (81 13 %; *P* < 0.001; all responses were partial responses) and the lymph node response rate (93 versus 4 %; *P* < 0.001). The rate of 12-month overall survival was 92 % with idelalisib and 80 % with placebo (*P* = 0.02); median overall survival had not been reached at the time of the analysis (Fig. 5b).

The most common AEs were pyrexia, fatigue, and nausea; rates were 29, 24, and 24 %, respectively, with idelalisib + rituximab and 16, 27, and 21 % with placebo + rituximab. The most common grade 3/4 AEs with idelalisib + rituximab were diarrhea (4 %), pyrexia (3 %), and fatigue (3 %); rates were 0, 1, and 2 %, respectively, with placebo + rituximab. With idelalisib + rituximab, incidences of grade 3/4 anemia, neutropenia, and thrombocytopenia were 5, 34, and 10 %, respectively, compared with 14, 22, and 16 % for placebo + rituximab. Transaminase elevations were more common with idelalisib + rituximab (all grade, 35 %; grade 3/4, 5 %) than with placebo + rituximab (all grade, 19 %; grade 3/4, 1 %); however, no patients discontinued therapy for these laboratory abnormalities. AE-related discontinuations were infrequent in both groups (idelalisib + rituximab, 8 % (mainly gastrointestinal and skin disorders); placebo + rituximab, 10 % (mainly infections and respiratory disorders)). Although long-term use needs to be evaluated, these data suggest that idelalisib + rituximab may be a treatment option for this difficult-to-treat population that is less able to undergo standard chemotherapy. A phase 2 study comparing the safety and efficacy of idelalisib monotherapy with idelalisib + rituximab in elderly patients (≥65 years of age) with previously untreated CLL or SLL is currently underway (NCT01203930).

## Conclusions

Chronic lymphocytic leukemia is most common in older patients, with approximately 70 % of diagnoses in patients ≥65 years of age [6], and first treatment typically occurs at an advanced age when many patients have multiple comorbidities [4, 5, 7, 8]. Although age and comorbidity are recognized as important considerations in prognostic assessment and choice of therapy, historically, clinical trials have excluded elderly and unfit patients because of legitimate ethical concerns regarding potential toxic effects in vital organs and worsening of comorbidity. However, with the development of oral therapies that are relatively nontoxic, CLL oncology is at the cusp of a paradigm shift whereby clinical trials will specifically address outcomes in elderly and unfit patients, who are more representative of the general CLL population.

Whereas in younger and/or fit patients with CLL, aggressive chemoimmunotherapy is recommended to achieve CR and prolong survival [10], such regimens are often poorly tolerated by elderly and/or unfit patients, necessitating an alternative approach. Although the NCCN guidelines recommend alternative anti-CD20 monoclonal antibody therapy regimens as first-line therapy in elderly and/or unfit patients [10], there have been few clinical trials of these therapies in this population. Ibrutinib, a BTK inhibitor, and idelalisib, a PI3K inhibitor, are the first of a new generation of agents to receive approval for treatment of CLL; several other kinase inhibitors, including fostamatinib, are in clinical development. These agents target BCR signaling, which plays a key role in B-cell processes fundamental to tumor growth, including B-cell proliferation and survival, and offers an important therapeutic target in CLL. Preclinical data for idelalisib, which is selective for the PI3Kδ isoform, provide a potential mechanism of action for clinical activity without toxicities associated with effects in nonhematopoietic cells. Recently reported trials of ibrutinib and idelalisib support the usefulness of kinase inhibitors as treatment options in elderly and unfit patients. The many ongoing trials will help address unmet need in this difficult-to-treat population by defining rates of CR and further informing optimal treatment strategies.

## Abbreviations

AEs: Adverse events
BCR: B-cell receptor
BTK: Bruton’s tyrosine kinase
CCI: Charlson comorbidity index
CIRS: Cumulative Illness Rating Scale
CLL: Chronic lymphocytic leukemia
CR: Complete response
ECOG: Eastern Cooperative Oncology Group
FCR: Rituximab with fludarabine and cyclophosphamide
ICED: Index of Coexistent Disease
NCCN: National Comprehensive Cancer Network
NCI: National Cancer Institute
NHL: Non-Hodgkin lymphoma
PFS: Progression-free survival
PI3K: Phosphatidylinositol 3-kinase
SEER: NCI surveillance, epidemiology, and end results
SYK: Spleen tyrosine kinase
SLL: Small lymphocytic lymphoma

## References

[1] Lobetti-Bodoni C, Bertoni F, Stussi G, Cavalli F, Zucca E (2013). The changing paradigm of chronic lymphocytic leukemia management. Eur J Intern Med.

[2] Hallek M, Pflug N (2010). Chronic lymphocytic leukemia. Ann Oncol.

[3] American Cancer Society. Cancer facts & figures 2015. Available at: http://www.cancer.org/acs/groups/content/@editorial/documents/document/acspc-044552.pdf. Accessed March 31, 2015.

[4] Hayat MJ, Howlader N, Reichman ME, Edwards BK (2007). Cancer statistics, trends, and multiple primary cancer analyses from the Surveillance, Epidemiology, and End Results (SEER) Program. Oncologist.

[5] Smith A, Howell D, Patmore R, Jack A, Roman E (2011). Incidence of haematological malignancy by sub-type: a report from the Haematological Malignancy Research Network. Br J Cancer.

[6] Gribben JG (2010). Chronic lymphocytic leukemia: planning for an aging population. Expert Rev Anticancer Ther.

[7] National Cancer Institute. SEER Cancer Statistics Review, 1975–2011. Available at: http://seer.cancer.gov/archive/csr/1975_2011/. Accessed March 31, 2015.

[8] Thurmes P, Call T, Slager S, Zent C, Jenkins G, Schwager S (2008). Comorbid conditions and survival in unselected, newly diagnosed patients with chronic lymphocytic leukemia. Leuk Lymphoma.

[9] Hallek M, Cheson BD, Catovsky D, Caligaris-Cappio F, Dighiero G, Dohner H (2008). Guidelines for the diagnosis and treatment of chronic lymphocytic leukemia: a report from the International Workshop on Chronic Lymphocytic Leukemia updating the National Cancer Institute-Working Group 1996 guidelines. Blood.

[10] National Comprehensive Cancer Network. NCCN Clinical Practice Guidelines in Oncology: Non-Hodgkin’s Lymphomas (version 1.2015). Available at: http://www.nccn.org/professionals/physician_gls/f_guidelines.asp#site.

[11] Smolewski P, Witkowska M, Korycka-Wolowiec A (2013). New insights into biology, prognostic factors, and current therapeutic strategies in chronic lymphocytic leukemia. ISRN Oncol.

[12] Cuthill K, Devereux S (2013). How I treat patients with relapsed chronic lymphocytic leukaemia. Br J Haematol.

[13] Hallek M, Fischer K, Fingerle-Rowson G, Fink AM, Busch R, Mayer J (2010). Addition of rituximab to fludarabine and cyclophosphamide in patients with chronic lymphocytic leukaemia: a randomised, open-label, phase 3 trial. Lancet.

[14] Gribben JG (2010). How I, treat CLL up front. Blood.

[15] Shvidel L, Shtalrid M, Bairey O, Rahimi-Levene N, Lugassy G, Shpilberg O (2003). Conventional dose fludarabine-based regimens are effective but have excessive toxicity in elderly patients with refractory chronic lymphocytic leukemia. Leuk Lymphoma.

[16] Hallek M (2013). Chronic lymphocytic leukemia: 2013 update on diagnosis, risk stratification and treatment. Am J Hematol.

[17] Robak T, Dmoszynska A, Solal-Celigny P, Warzocha K, Loscertales J, Catalano J (2010). Rituximab plus fludarabine and cyclophosphamide prolongs progression-free survival compared with fludarabine and cyclophosphamide alone in previously treated chronic lymphocytic leukemia. J Clin Oncol.

[18] Gozzetti A, Candi V, Fabbri A, Schiattone L, Cencini E, Lauria F (2014). Chemoimmunotherapy with oral low-dose fludarabine, cyclophosphamide and rituximab (old-FCR) as treatment for elderly patients with chronic lymphocytic leukaemia. Leuk Res.

[19] Kolibaba KS, Sterchele JA, Joshi AD, Forsyth M, Alwon E, Beygi H (2013). Demographics, treatment patterns, safety, and real-world effectiveness in patients aged 70 years and over with chronic lymphocytic leukemia receiving bendamustine with or without rituximab: a retrospective study. Ther Adv Hematol.

[20] Shanafelt TD, Lin T, Geyer SM, Zent CS, Leung N, Kabat B (2007). Pentostatin, cyclophosphamide, and rituximab regimen in older patients with chronic lymphocytic leukemia. Cancer.

[21] Shanafelt T, Lanasa MC, Call TG, Beaven AW, Leis JF, LaPlant B (2013). Ofatumumab-based chemoimmunotherapy is effective and well tolerated in patients with previously untreated chronic lymphocytic leukemia (CLL). Cancer.

[22] Goede V, Fischer K, Busch R, Engelke A, Eichhorst B, Wendtner CM (2014). Obinutuzumab plus chlorambucil in patients with CLL and coexisting conditions. N Engl J Med.

[23] Oken MM, Creech RH, Tormey DC, Horton J, Davis TE, McFadden ET (1982). Toxicity and response criteria of the Eastern Cooperative Oncology Group. Am J Clin Oncol.

[24] Karnofsky DA, Burchenal JH, MacLeod CM (1949). The clinical evaluation of chemotherapeutic agents in cancer. Evaluation of chemotherapeutic agents.

[25] Charlson ME, Pompei P, Ales KL, MacKenzie CR (1987). A new method of classifying prognostic comorbidity in longitudinal studies: development and validation. J Chronic Dis.

[26] de Groot V, Beckerman H, Lankhorst GJ, Bouter LM (2003). How to measure comorbidity. A critical review of available methods. J Clin Epidemiol.

[27] Sorror ML, Maris MB, Storb R, Baron F, Sandmaier BM, Maloney DG (2005). Hematopoietic cell transplantation (HCT)-specific comorbidity index: a new tool for risk assessment before allogeneic HCT. Blood.

[28] Havlik RJ, Yancik R, Long S, Ries L, Edwards B (1994). The National Institute on Aging and the National Cancer Institute SEER collaborative study on comorbidity and early diagnosis of cancer in the elderly. Cancer.

[29] Yancik R, Havlik RJ, Wesley MN, Ries L, Long S, Rossi WK (1996). Cancer and comorbidity in older patients: a descriptive profile. Ann Epidemiol.

[30] Linn BS, Linn MW, Gurel L (1968). Cumulative illness rating scale. J Am Geriatr Soc.

[31] Greenfield S, Apolone G, McNeil BJ, Cleary PD (1993). The importance of co-existent disease in the occurrence of postoperative complications and one-year recovery in patients undergoing total hip replacement. Comorbidity and outcomes after hip replacement. Med Care.

[32] CLLTrialists’ Collaborative Group (2012). Systematic review of purine analog treatment for chronic lymphocytic leukemia: lessons for future trials. Haematologica.

[33] Eichhorst B, Goede V, Hallek M (2009). Treatment of elderly patients with chronic lymphocytic leukemia. Leuk Lymphoma.

[34] Danilov AV (2013). Targeted therapy in chronic lymphocytic leukemia: past, present, and future. Clin Ther.

[35] Wu M, Akinleye A, Zhu X (2013). Novel agents for chronic lymphocytic leukemia. J Hematol Oncol.

[36] Rickert RC (2013). New insights into pre-BCR and BCR signalling with relevance to B cell malignancies. Nat Rev Immunol.

[37] Lam KP, Kuhn R, Rajewsky K (1997). In vivo ablation of surface immunoglobulin on mature B cells by inducible gene targeting results in rapid cell death. Cell.

[38] Wiestner A (2012). Emerging role of kinase-targeted strategies in chronic lymphocytic leukemia. Blood.

[39] Herman SE, Gordon AL, Hertlein E, Ramanunni A, Zhang X, Jaglowski S (2011). Bruton tyrosine kinase represents a promising therapeutic target for treatment of chronic lymphocytic leukemia and is effectively targeted by PCI-32765. Blood.

[40] Bajpai UD, Zhang K, Teutsch M, Sen R, Wortis HH (2000). Bruton’s tyrosine kinase links the B cell receptor to nuclear factor kappaB activation. J Exp Med.

[41] Shinners NP, Carlesso G, Castro I, Hoek KL, Corn RA, Woodland RT (2007). Bruton’s tyrosine kinase mediates NF-kappa B activation and B cell survival by B cell-activating factor receptor of the TNF-R family. J Immunol.

[42] Fruman DA (2004). Phosphoinositide 3-kinase and its targets in B-cell and T-cell signaling. Curr Opin Immunol.

[43] Puri KD, Gold MR (2012). Selective inhibitors of phosphoinositide 3-kinase delta: modulators of B-cell function with potential for treating autoimmune inflammatory diseases and B-cell malignancies. Front Immunol.

[44] Srinivasan L, Sasaki Y, Calado DP, Zhang B, Paik JH, DePinho RA (2009). PI3 kinase signals BCR-dependent mature B cell survival. Cell.

[45] Herman SE, Gordon AL, Wagner AJ, Heerema NA, Zhao W, Flynn JM (2010). Phosphatidylinositol 3-kinase-delta inhibitor CAL-101 shows promising preclinical activity in chronic lymphocytic leukemia by antagonizing intrinsic and extrinsic cellular survival signals. Blood.

[46] Tan SL, Liao C, Lucas MC, Stevenson C, DeMartino JA (2013). Targeting the SYK-BTK axis for the treatment of immunological and hematological disorders: recent progress and therapeutic perspectives. Pharmacol Ther.

[47] Robak T, Robak P (2013). BCR signaling in chronic lymphocytic leukemia and related inhibitors currently in clinical studies. Int Rev Immunol.

[48] de Rooij MF, Kuil A, Geest CR, Eldering E, Chang BY, Buggy JJ (2012). The clinically active BTK inhibitor PCI-32765 targets B-cell receptor- and chemokine-controlled adhesion and migration in chronic lymphocytic leukemia. Blood.

[49] Ponader S, Chen SS, Buggy JJ, Balakrishnan K, Gandhi V, Wierda WG (2012). The Bruton tyrosine kinase inhibitor PCI-32765 thwarts chronic lymphocytic leukemia cell survival and tissue homing in vitro and in vivo. Blood.

[50] Vanhaesebroeck B, Welham MJ, Kotani K, Stein R, Warne PH, Zvelebil MJ (1997). P110delta, a novel phosphoinositide 3-kinase in leukocytes. Proc Natl Acad Sci U S A.

[51] Friedberg JW, Sharman J, Sweetenham J, Johnston PB, Vose JM, Lacasce A (2010). Inhibition of Syk with fostamatinib disodium has significant clinical activity in non-Hodgkin lymphoma and chronic lymphocytic leukemia. Blood.

[52] Herman SE, Barr PM, McAuley EM, Liu D, Wiestner A, Friedberg JW (2013). Fostamatinib inhibits B-cell receptor signaling, cellular activation and tumor proliferation in patients with relapsed and refractory chronic lymphocytic leukemia. Leukemia.

[53] Curran E, Smith SM (2014). Phosphoinositide 3-kinase inhibitors in lymphoma. Curr Opin Oncol.

[54] Akinleye A, Avvaru P, Furqan M, Song Y, Liu D (2013). Phosphatidylinositol 3-kinase (PI3K) inhibitors as cancer therapeutics. J Hematol Oncol.

[55] Burger JA (2014). Bruton’s tyrosine kinase (BTK) inhibitors in clinical trials. Curr Hematol Malig Rep.

[56] Akinleye A, Chen Y, Mukhi N, Song Y, Liu D (2013). Ibrutinib and novel BTK inhibitors in clinical development. J Hematol Oncol.

[57] Burke RT, Meadows S, Loriaux MM, Currie KS, Mitchell SA, Maciejewski P (2014). A potential therapeutic strategy for chronic lymphocytic leukemia by combining Idelalisib and GS-9973, a novel spleen tyrosine kinase (Syk) inhibitor. Oncotarget.

[58] Hendriks RW, Yuvaraj S, Kil LP (2014). Targeting Bruton’s tyrosine kinase in B cell malignancies. Nat Rev Cancer.

[59] Vogler M, Dinsdale D, Dyer MJ, Cohen GM (2013). ABT-199 selectively inhibits BCL2 but not BCL2L1 and efficiently induces apoptosis of chronic lymphocytic leukaemic cells but not platelets. Br J Haematol.

[60] Souers AJ, Leverson JD, Boghaert ER, Ackler SL, Catron ND, Chen J (2013). ABT-199, a potent and selective BCL-2 inhibitor, achieves antitumor activity while sparing platelets. Nat Med.

[61] O’Brien S, Furman RR, Coutre SE, Sharman JP, Burger JA, Blum KA (2014). Ibrutinib as initial therapy for elderly patients with chronic lymphocytic leukaemia or small lymphocytic lymphoma: an open-label, multicentre, phase 1b/2 trial. Lancet Oncol.

[62] Furman RR, Sharman JP, Coutre SE, Cheson BD, Pagel JM, Hillmen P (2014). Idelalisib and rituximab in relapsed chronic lymphocytic leukemia. N Engl J Med.

